# Transient, activity dependent inhibition of transmitter release from low threshold afferents mediated by GABA_A_ receptors in spinal cord lamina III/IV

**DOI:** 10.1186/s12990-015-0067-5

**Published:** 2015-10-13

**Authors:** Chiara Betelli, Amy B. MacDermott, Rita Bardoni

**Affiliations:** Department of Biomedical, Metabolic and Neural Sciences, University of Modena and Reggio Emilia, Via Campi, 287, 41125 Modena, Italy; Departments of Physiology and Cellular Biophysics, Neuroscience, Columbia University, 630 W. 168th Street, New York, NY 10032 USA

**Keywords:** Presynaptic inhibition, Dorsal horn, GABA_A_

## Abstract

**Background:**

Presynaptic GABA_A_ receptors (GABA_A_Rs) located on central terminals of low threshold afferent fibers are thought to be involved in the processing of touch and possibly in the generation of tactile allodynia in chronic pain. These GABA_A_Rs mediate primary afferent depolarization (PAD) and modulate transmitter release. The objective of this study was to expand our understanding of the presynaptic inhibitory action of GABA released onto primary afferent central terminals following afferent stimulation.

**Results:**

We recorded evoked postsynaptic excitatory responses (eEPSCs and eEPSPs) from lamina III/IV neurons in spinal cord slices from juvenile rats (P17–P23, either sex), while stimulating dorsal roots. We investigated time and activity dependent changes in glutamate release from low threshold A fibers and the impact of these changes on excitatory drive. Blockade of GABA_A_Rs by gabazine potentiated the second eEPSC during a train of four afferent stimuli in a large subset of synapses. This resulted in a corresponding increase of action potential firing after the second stimulus. The potentiating effect of gabazine was due to inhibition of endogenously activated presynaptic GABA_A_Rs, because it was not prevented by the blockade of postsynaptic GABA_A_Rs through intracellular perfusion of CsF. Exogenous activation of presynaptic GABA_A_Rs by muscimol depressed evoked glutamate release at all synapses and increased paired pulse ratio (PPR).

**Conclusions:**

These observations suggest that afferent driven release of GABA onto low threshold afferent terminals is most effective following the first action potential in a train and serves to suppress the initial strong excitatory drive onto dorsal horn circuitry.

## Background

Mechanical stimulation of the skin activates low threshold, cutaneous mechanoreceptors, which in turn activates their spinal and medullary synaptic targets. However, stimulation of low threshold afferents also drives feedback inhibition within the spinal cord that depresses subsequent transmitter release from the mechanoreceptor central terminals. This presynaptic inhibition is caused by depolarization of the mechanoreceptor central terminals [1] termed primary afferent depolarization (PAD). The inhibitory feedback effect has been reported for a wide variety of tactile modalities and therefore tactile receptor types [2, 3]. PAD and the associated presynaptic inhibition of afferent terminals in the spinal cord is, in the most classical view, mediated by GABA and GABA_A_ receptors [4, 5], although other transmitters and receptors are likely to be involved as well [6, 7]. It has been suggested that an important physiological role for this inhibitory control of transmitter release from cutaneous mechanoreceptors is that it allows central suppression of tactile input [8] or spatial focusing of cutaneous input in the spinal cord [9].

De Koninck and Henry [10] directly investigated the contribution of presynaptic GABA_A_ receptors to strong paired pulse depression between Aβ (group II) and Aδ (group III) hair afferents and lamina III/IV neurons in vivo. They found that stimulation of a single Aβ afferent produced a burst of action potential activity in the dorsal horn neuron under study and that, when pairs of action potentials were applied to a single afferent, the second burst of activity was considerably smaller than the first. The GABA_A_ receptor antagonist, bicuculline, was substantially and reversibly able to diminish the depression of the second response to Aβ fiber stimulation or mechanical hair stimulation indicating a presynaptic action. In contrast, bicuculline enhanced both the first and second responses to Aδ fiber stimulation, suggesting a postsynaptic site of control [10].

We have used an in vitro preparation, spinal cord slices from postnatal day 17–23 old rats, to investigate the impact of synaptic depression at A fiber terminals, especially the component mediated by GABA_A_ receptor activation, on synaptic excitation of lamina III/IV neurons. Using both synaptic and agonist activation of receptors on the presynaptic terminals of low threshold fibers, we have been able to identify transient, activity-dependence of GABA modulated release and to demonstrate its impact on excitatory drive in the postsynaptic lamina III/IV neurons.

## Results

Patch-clamp recordings were obtained from a total of 83 lamina III/IV neurons from juvenile (P17–23) rats, identified as being ventral to the translucent lamina II layer and larger than the lamina II neurons. All recorded cells were located at a distance of 200–300 μm from the white matter, corresponding to the position of laminae III/IV in rat lumbar spinal cord [11, 12]. Evoked excitatory postsynaptic currents (eEPSCs) mediated by A fibers (including mainly Aβ and possibly some Aδ fibers) were identified at the beginning of each experiment by stimulating the dorsal root at high frequency (20 Hz) and low stimulation intensity (mostly between 25 and 50 μA) [12–15]. All analysis of eEPSCs (from a total of 73 neurons) was performed on monosynaptic responses, identified as synaptic currents exhibiting no failures and a constant latency (variation within 0.5 ms) [12].

### Endogenous modulation of transmitter release

**Fig. 1:**
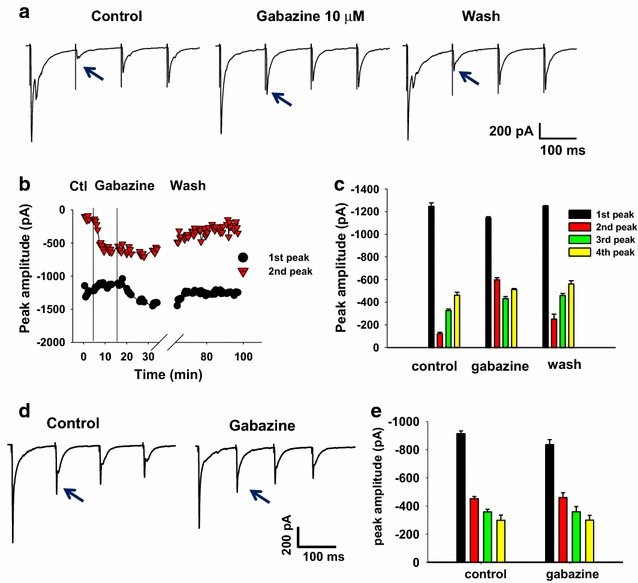
Endogenous, activity dependent activation of presynaptic GABA_A_ receptors. **a** Averaged evoked EPSCs (from 5 to 6 traces), obtained by repetitively stimulating the primary afferent fibers (four pulses, 10 Hz), while recording from a lamina III/IV neuron. In this example, the second eEPSC peak (marked by the *arrow*) is smaller than the first, third and fourth peaks. The GABA_A_ receptor antagonist, gabazine, caused a substantial increase of the second peak that is reversible in wash. **b** Time course of the effect of gabazine on the first (*circles*) and second peak (*triangles*), from the example shown in **a**. Parallel *vertical bars* indicate the time course during which gabazine was added to the bath. **c** Mean amplitude values of the four eEPSC peaks from the same recording as shown in **a**, determined under the different experimental conditions. **d** Evoked EPSCs recorded from a different lamina III/IV neuron (averaged EPSCs from five consecutive traces). Under control conditions, the eEPSC peaks show a different pattern of amplitude changes compared to the neuron in **a**, with the second peak being larger than the third and fourth. In this case the second peak amplitude is not altered by gabazine. **e** Mean peak amplitudes are plotted from the same data as shown in **d**

Dorsal root potential (DRP) recordings provide a way to measure the time course of PAD, which in turn reflects the time course of transmitter activation of the presynaptic terminals [5]. In vitro and in vivo DRP measurements indicate that following a single stimulus to the dorsal root, DRP associated with presynaptic GABA_A_ receptor activation peaks in about 50–100 ms and lasts for about 1 s [10, 16]. Based on this time course, we applied trains of four stimuli at 10–20 Hz to the dorsal roots attached to our spinal cord slices to investigate afferent-driven presynaptic inhibition of glutamate release from primary afferents, mediated by GABAergic interneurons. Monosynaptic eEPSCs recorded from lamina III/IV neurons always exhibited synaptic depression to the second, third and fourth stimuli, as shown in two sample recordings obtained from two different neurons using a 10 Hz stimulation train (Fig. 1a, d, control panels). Only a subset of those synapses showed sensitivity to the GABA_A_ antagonist gabazine (Fig. 1a). The complete experimental time course of the effect of gabazine on the first and second peaks in this example is shown in Fig. 1b. As gabazine washed onto the spinal cord slice, indicated by the vertical lines, the second peak was strongly enhanced while the first peak showed little change. The average amplitudes of all four peaks before, during and after gabazine are shown in Fig. 1c. It is clear that the dominant effect of gabazine is on the second peak. In contrast, the example of Fig. 1d and e shows that the second, third and fourth peak amplitudes were all depressed under control conditions, but were insensitive to gabazine.

**Fig. 2:**
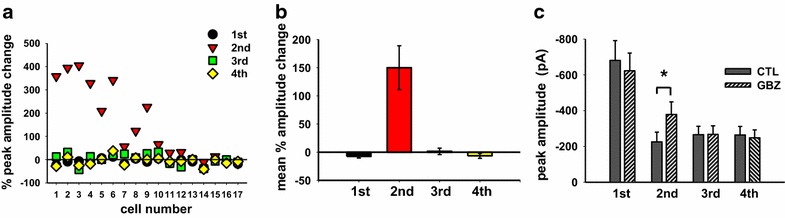
Gabazine enhances the second eEPSC peak in a train of four pulses. **a** Percentage changes of the eEPSC mean peak amplitudes (determined by averaging five traces in control and five traces in gabazine) in a sample of 17 lamina III/IV neurons (same experiment illustrated in Fig. 1). Cells numbered 1–10 showed a significant effect of gabazine on the second peak. **b** Mean values of percentage changes for each peak in the 17 neuron sample. **c** Mean absolute peak amplitudes in control and gabazine: the antagonist produces a significant increase only in the second eEPSC peak (paired t test, p = 0.013, n = 17)

Analysis of the impact of gabazine on A fiber-induced eEPSC trains is shown in Fig. 2. The percent change in peak amplitude in response to gabazine for each of the four eEPSCs at each synapse tested is graphed in Fig. 2a. It is apparent that, on a cell by cell basis, only the amplitude of the second response is significantly enhanced by gabazine and only in a subpopulation of neurons (n = 10/17). Averaged across all neurons, the only significant change with gabazine was in the amplitude of the second peak when viewed either as mean percent change in eEPSC amplitude (Fig. 2b) or absolute amplitude (Fig. 2c; paired *t* test, p = 0.013, n = 17). None of the other peaks were significantly affected by gabazine.

**Fig. 3:**
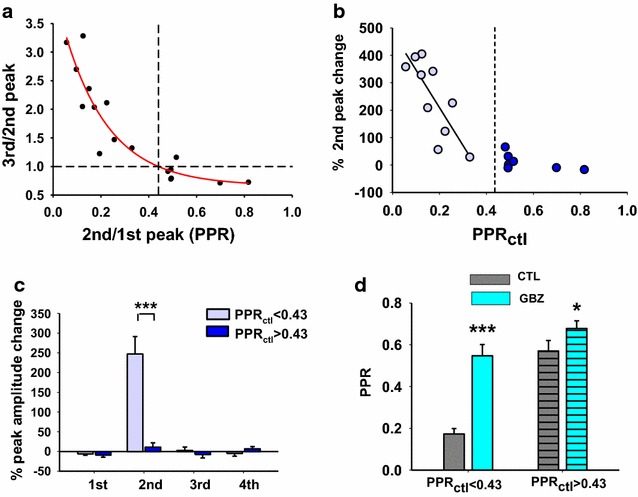
Synapses exhibiting different patterns of synaptic depression have different sensitivity to gabazine. **a** Plotting of third/second peak ratios, determined from the 17 cells shown in Fig. 2, as function of the PPR (second/first peak ratio). The single exponential function best fitting the data (y = y_0_ + a * exp(−b * x)) is represented by the *solid line* (r = 0.92; rsqr = 0.86; y = 1 for x = 0.43). **b**, **c** Effects of gabazine on the second eEPSC peak **b** and on all peaks **c**, as a function of PPR_ctl_. **b** The second peak percentage change is strongly correlated with PPR when this is less than 0.43 (Pearson correlation test, p = 0.005, n = 10). **c** Only the second peak percentage change exhibits a significant difference between synapses with PPR <0.43 and PPR >0.43 (unpaired t test, p < 0.001, n = 10 and 7, respectively). **d** Mean values of PPR in control and gabazine, plotted for the two populations of synapses. Both sets of synapses exhibit a significant increase of PPR in gabazine, although those with PPR <0.43 reach a higher level of significance (PPR <0.43: paired t test, p < 0.001, n = 10; PPR >0.43: paired t test, p = 0.037, n = 7)

Prior to application of gabazine, two different patterns of synaptic depression were observed in this 17 cell sample. In the first group of synapses, the second peak was more strongly depressed than the subsequent third and fourth responses (Fig. 1a). In the second group (Fig. 1d), the second peak was less depressed and its amplitude was larger than the third and fourth peaks. In order to obtain a quantitative representation of the two groups, the ratio of the third peak over the second peak was plotted as a function of conventional paired pulse ratio (PPR, i.e. the second peak over the first) (Fig. 3a). This provided an indicator of how similar or different the second and third peaks were. Synapses belonging to the first group with a third/second peak ratio >1 tended to have lower PPRs (<0.43, determined from the single exponential function fitting the data), while synapses from the second group (third/second peak <1) had higher PPRs (>0.43).

We considered possible underlying mechanisms for these two patterns of synaptic depression observed during four pulses trains. They were not due to activation of different afferent types because we did not find a clear correlation between stimulation intensities and PPR values (data not shown). As suggested by the data in Fig. 1, the two patterns of depression could be due to a different impact of GABA_A_ receptor mediated presynaptic inhibition. To test this hypothesis, we plotted the percent change in amplitude of the second peak after exposure to gabazine as a function of control PPR for each individual neuron tested (Fig. 3b). Most synapses exhibiting PPRs <0.43 were sensitive to gabazine (n = 9/10), while synapses with PPR >0.43 were mostly insensitive (only 1 cell out of 7 showed a significant effect of gabazine). When changes induced by gabazine on each of the four peak amplitudes were plotted separately for synapses with PPR <0.43 and PPR >0.43, we observed a strong enhancement of the second response (and only the second response) for synapses with PPR <0.43 that was significantly larger than the increase observed at synapses with PPR >0.43 (Fig. 3c; t test, p < 0.001, n = 10 and 7, respectively). Similarly, when the gabazine effect on PPR was directly compared within the two populations of synapses, synapses with PPR <0.43 showed a much higher impact of the GABA_A_ antagonist (Fig. 3d, paired t test, p < 0.001, n = 10), although a modest but still significant enhancement of PPR by gabazine in the PPR >0.43 group was also detected (Fig. 3d, paired t test, p = 0.037, n = 7). These data demonstrate that at PPR <0.43, the strong afferent driven depression of the second peak is mediated by GABA and GABA_A_ receptors while the subsequent depression of peaks three and four is not mediated by GABA and GABA_A_ receptors.

**Fig. 4:**
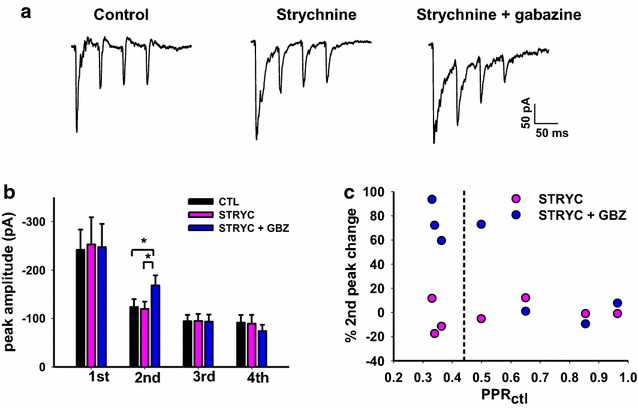
Glycine receptors do not contribute to presynaptic inhibition of transmitter release. **a** Example of the effects of 300 nM strychnine and strychnine plus 10 μM gabazine on a train of four eEPSCs recorded from a lamina III/IV neuron (averaged eEPSCs from 5 to 6 consecutive traces in each condition). **b** Mean EPSC peak amplitudes in control, strychnine and strychnine plus gabazine are plotted for each of the four synaptic events, from a sample of seven lamina III/IV neurons. While strychnine alone is not effective, addition of gabazine causes a significant increase of the second peak (repeated measures ANOVA, Tukey post-test, p < 0.05, n = 7). **c** The increase of the eEPSC second peak in gabazine is related to PPR_ctl_, as shown in Fig. 3

Lamina III/IV neurons receive inhibitory synaptic input predominantly mediated by glycine rather that GABA [17, 18]. This provides evidence that glycine is released as the dominant inhibitory transmitter in lamina III/IV and raises the possibility that glycine could also be acting presynaptically. Therefore, we tested whether the presynaptic inhibition in our studies might be mediated by glycine as well as GABA in some instances. Application of 300 nM strychnine, however, did not significantly affect the amplitude of the four eEPSCs in a 20 Hz stimulation train (Fig. 4a, b). Subsequent addition of gabazine still produced a significantly increased amplitude of the second EPSC when comparing average peak amplitudes in control and the two drugs (repeated measures ANOVA, Tukey post test, p < 0.05, n = 7; Fig. 4b). Figure 4c shows within-cell comparison of strychnine and strychnine plus gabazine on PPR when plotted as a function of control PPR. The lack of effect of strychnine in all seven cases suggests that presynaptic glycine receptors are either not expressed on terminals of primary afferent fibers or are not endogenously activated under our experimental conditions. In about half of the synapses tested, those with lower PPRs, subsequent application of gabazine enhanced the second peak (Fig. 4c).

### Postsynaptic GABA_A_ do not account for synaptic depression

The strong enhancement by gabazine of the second peak in a train of four, together with the associated change in PPR, suggests that the relevant GABA_A_ receptors are expressed on the central terminals of presynaptic A fibers. Nevertheless, it was important to test whether activation of GABAergic inhibitory postsynaptic currents (IPSCs) temporally overlapping with our eEPSCs might contribute to our results. It was possible, for example, that the second eEPSC in the train might be shunted by an underlying IPSC (eIPSC) driven by polysynaptic input, as was the case for synaptic depression with Aδ fibers in [10]. Furthermore, the amplitude and kinetics of glutamatergic EPSCs, recorded in voltage-clamp at membrane potentials close to E_Cl_, could be significantly affected by the opening of GABA_A_ channels [19, 20].

Intracellularly applied fluoride ions have been reported to block GABA_A_ receptors due to their low permeability through the Cl channel [21, 22]. Therefore, we used a CsF based intracellular solution to block postsynaptic GABA_A_ receptors while not affecting presynaptic receptors, thus allowing us to test whether the gabazine effect enhancing the second peak was due to a postsynaptic action.

**Fig. 5:**
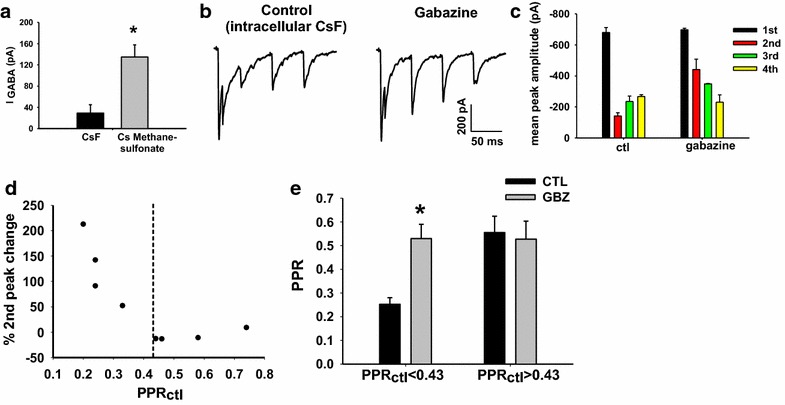
Gabazine-induced increase of PPR is not an indirect effect of postsynaptic GABA_A_ receptor activation. **a** Perfusion of lamina III/IV neurons with a CsF-based intracellular solution inhibits muscimol-induced current compared to that observed in intracellular Cs-methanesulfonate. **b**, **c** The presence of intracellular CsF does not prevent the effect of gabazine on the second eEPSC amplitude in a lamina III/IV neuron. **b** averaged traces from five consecutive EPSCs recorded in control and in gabazine 10 μM. **c** Mean peak amplitudes of the four EPSCs recorded from the neuron represented in **b**. **d** Population data (from a total of eight cells) comparing the change in the second peak as a function of control PPR. **e** Shows the PPR plotted before and after gabazine when control PPR is <0.43 or >0.43. Only at synapses with PPRctl <0.43 gabazine causes a significant increase of PPR (paired t test, p = 0.006, n = 4)

We first tested the efficacy of intracellular CsF in blocking GABA_A_ receptors in our spinal cord preparation by recording currents activated by the selective GABA_A_ receptor agonist, muscimol, at −30 mV, in a sample of 10 lamina III/IV neurons. Five neurons were recorded with a normal Cs-methanesulfonate intracellular solution while the other five were intracellularly perfused with CsF. Under these conditions, CsF strongly reduced the response to 2 µM muscimol (Fig. 5a). Although a residual muscimol-induced current was still present in CsF, the average current amplitude was significantly decreased by 78 % compared to the current recorded in Cs-methanesulfonate (unpaired t test, p = 0.02, n = 10; Fig. 5a).

Ten μM gabazine applied during repetitive dorsal root stimulation while recording from neurons perfused intracellularly with CsF solution was still able to induce a potentiation of the second eEPSC at synapses with PPR <0.43. Figure 5b and c show an example of this experiment. As was true without intracellular fluoride, only the second response amplitude out of the four in the train was enhanced by gabazine (Fig. 5b, c). All four neurons studied with control PPR <0.43 had a significant change in amplitude of the second response in gabazine (Fig. 5d) and a significantly enhanced PPR (paired t test, p = 0.006; Fig. 5e). The four neurons with PPR >0.43 showed no change in the second response amplitude and correspondingly no change in PPR (paired t test, p > 0.05, n = 4; Fig. 5e).

### Impact of endogenous modulation of transmitter release on synaptically driven action potential firing

**Fig. 6:**
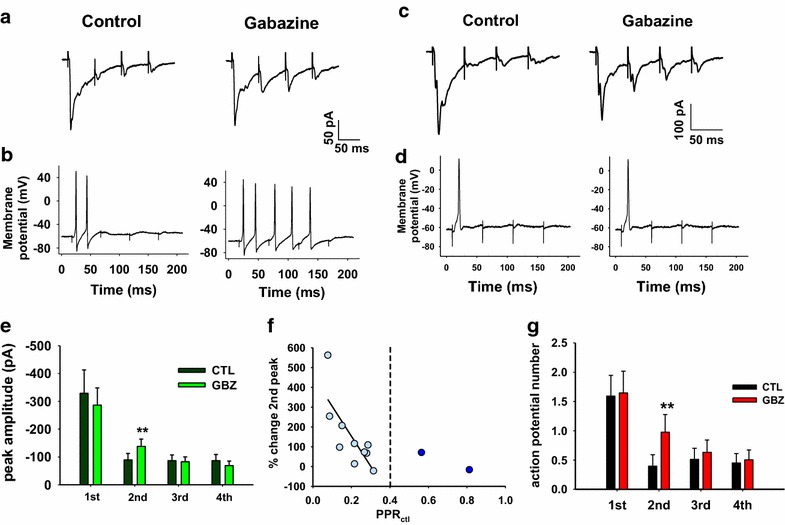
Gabazine enhances action potential firing to the second stimulus in a four stimulus train in a subpopulation of synapses. **a**, **b** Example of eEPSC/P traces recorded from a lamina III/IV neuron in voltage (**a** average of five consecutive traces) and current clamp (**b** individual, representative traces), evoked by repetitive stimulation (four pulses, 20 Hz). Gabazine increases both the second eEPSC amplitude and action potential firing. **c**, **d** Example of a different neuron where gabazine produces the increase of the second eEPSC peak, without altering action potential firing (**c** average of five consecutive traces; **d** individual, representative traces). **e** Effect of gabazine on mean absolute peak amplitudes from a sample of 12 neurons. Gabazine causes a significant increase of the second peak (paired t test; p = 0.007, n = 12). **f** Relation between the effect of gabazine on the second peak and PPR_ctl_. As shown in Fig. 3b, there is a strong correlation between the effect of gabazine on the second peak and PPR when it is less than 0.43 (Pearson correlation test, p = 0.008, n = 10). **g** Mean number of action potentials generated at each stimulus (average of 5–6 consecutive traces, same sample of 12 neurons shown in **e**, **f**). Action potential firing is significantly increased only at the second stimulus during gabazine, considering the whole sample of 12 lamina III/IV neurons (paired t test, p = 0.008, n = 12)

Thus far, we have demonstrated that presynaptic inhibition of glutamate release from central terminals of A fibers is a transient inhibition, only affecting the second response to a train of four stimuli applied at 10 Hz (Figs. 1, 2, 3). We next directly determined the consequences of this presynaptic inhibition by recording the evoked synaptic activity under current clamp conditions. We used trains of four stimuli at 20 Hz, allowing us to enhance synaptic summation and general excitatory drive of the postsynaptic neurons. We recorded responses to stimulation trains first in voltage clamp to allow us to identify gabazine sensitive synapses in our usual way and then in current clamp from the same neurons. Recordings were made from 12 lamina III/IV neurons (Fig. 6).

Most synapses in this experiment belonged to the low PPR (<0.43) population (n = 10/12; Fig. 6a, c, f). Application of gabazine induced a significant increase of the second eEPSC in most of the synaptic connections (9/12, Fig. 6a, c, f). The effect of gabazine on action potential firing recorded under current clamp at these same synapses was more heterogeneous. In four neurons in which a significant potentiation of the second eEPSC was observed, there was an increase in action potential firing, as shown in the example in Fig. 6a, b. In the remaining five neurons with gabazine potentiation of the second eEPSC, generation of action potentials was unaffected (Fig. 6c, d). Analyses performed on the peak amplitudes averaged across all 12 cells showed that gabazine induced a significant potentiation of the second eEPSC amplitude (paired t test; p = 0.007, n = 12; Fig. 6e) and a correspondingly significant increase of action potential firing to the second stimulus (paired t test, p = 0.008, n = 12; Fig. 6g). This suggests that in a subpopulation of lamina III/IV neurons, low threshold afferent input is subject to transient presynaptic inhibition mediated by GABA_A_ receptors that normally suppresses glutamate release and thus suppresses subsequent action potential firing.

### Transmitter release modulated by activation of presynaptic GABA_A_ receptors at all synapses

**Fig. 7:**
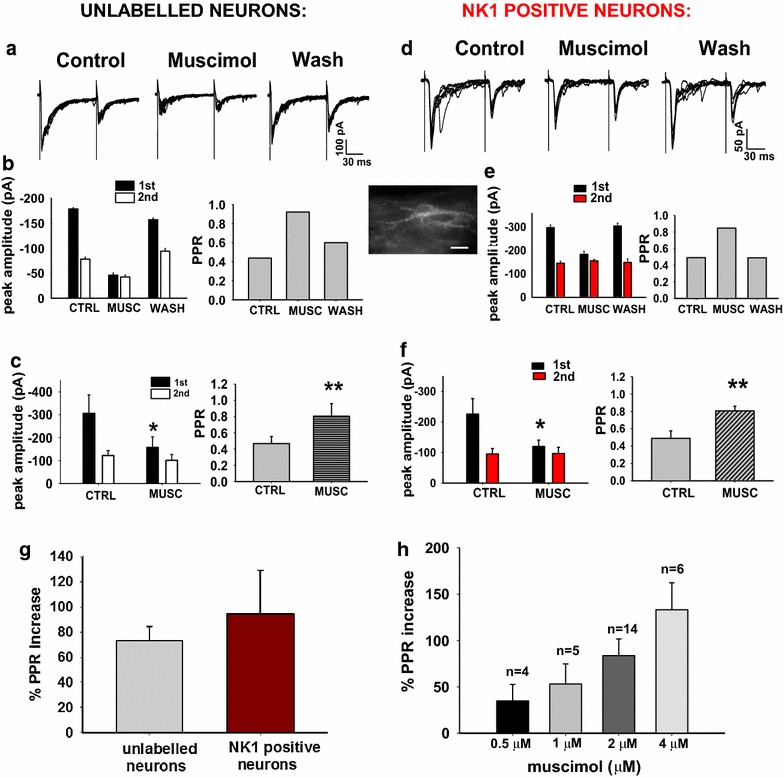
Presynaptic muscimol effect on EPSC amplitudes in lamina III/IV neurons, recorded with a paired pulse protocol. **a** Example of the inhibitory effect of 2 μM muscimol on 10 individual traces, recorded from an unidentified lamina III/IV neuron: both eEPSC peaks were reversibly depressed. **b** Mean peak amplitudes of the two peaks are shown from the same neuron as in **a**. The second/first peak ratio (PPR) was enhanced. **c** Muscimol caused a significant depression of the first eEPSC amplitude and a significant increase of PPR in a population of seven unlabeled neurons (paired t test: p = 0.013 and p = 0.004, respectively, n = 7). **d** Effect of muscimol on seven individual eEPSCs recorded from an NK1 receptor positive, lamina III/IV neuron (in the *inset*: fluorescence image of the neuron, *scale bar* 20 μm). **e** Muscimol application reversibly depressed the first eEPSC amplitude and increased PPR in this neuron. **f** In a sample of seven NK1+ neurons, muscimol significantly depressed the first eEPSC and enhanced PPR (paired t test, p = 0.02 and p = 0.007, respectively, n = 7). **g** Percentage change of PPR induced by muscimol in the two groups of neurons was not significantly different. **h** Mean PPR increase obtained at different muscimol concentrations. The number of lamina III/IV neurons tested at each concentration is reported above each *bar*

We have shown that afferent driven, GABA release onto primary afferent terminals depresses the second response to a train of stimuli but only in a subpopulation of the synapses tested. To establish whether this reflects a unique expression of GABA_A_ receptors only on the gabazine sensitive synaptic terminals, we tested for the presence of presynaptic GABA_A_ receptors with exogenous agonist. The GABA_A_ receptor agonist, muscimol, was applied for 5 min while recording monosynaptic eEPSCs from lamina III/IV neurons. Evoked EPSCs were recorded at −70 mV holding potential, applying a paired pulse protocol (100 ms interval) every 30 s (Fig. 7a). The average control PPR was 0.47 ± 0.1 (n = 7, Fig. 7a, c). Application of muscimol (2 μM) rapidly caused a reversible, large and significant depression of the first EPSC of all synapses tested (mean percentage depression: 50.3 ± 9.6; paired t test: p = 0.013, n = 7; Fig. 7a–c). A rapid, partial desensitization of presynaptic GABA_A_ receptors and a corresponding decrease over time of the muscimol effect on transmitter release have been described. Because we recorded most paired EPSCs during the steady-state phase of the muscimol response, we did not observe a substantial change in the depression of the first peak during the GABA_A_ agonist application. Average reduction of the second peak was small and not significant (mean percentage depression: 16.8 ± 15.3; paired t test: p > 0.05, n = 7). Mean PPR was significantly increased by muscimol (paired t test: p = 0.004, n = 7; Fig. 7b, c), suggesting a presynaptic site of action. Muscimol application also induced a variable change of holding current and a decrease of cell input resistance (data not shown), indicating that GABA_A_ receptors located on the dorsal horn neuron under study were also activated.

Subpopulations of neurons in lamina III/IV, but not in lamina II, express receptor for substance P, the NK1 receptor (NK1R). Some of these project to brainstem and thalamic areas of the brain [23]. Identification of NK1R expressing neurons (NK1R+) provides a way to study a less heterogeneous population of lamina III/IV neurons than recording with no neural marker. We have previously shown that with respect to primary afferent input, 70 % of the NK1R+ neurons in lamina III/IV receive only Aβ fiber input [12].

Pre-incubation of spinal cord slices in fluorescent TMR-Substance P allowed pre-identification of lamina III/IV neurons expressing NK1 receptors. Muscimol (2 μM) produced effects on eEPSC amplitudes (Fig. 7d, e) that were similar to the muscimol effect on non-identified neurons. The first peak was significantly depressed (mean percentage depression: 42.3 ± 6.2; paired t test, p = 0.02, n = 7), the second peak was not significantly affected (mean percentage change: 4.6 ± 12.9; paired t test: p > 0.05, n = 7) and mean PPR was significantly increased (paired t test, p = 0.007, n = 7) (Fig. 7e, f). Comparison of the increase in PPR produced by muscimol on lamina III/IV unidentified and NK1+ neurons did not reveal a significant difference (unpaired t test, p > 0.05, n = 14; Fig. 7g). Finally, the increase in PPR due to muscimol was greater with increasing doses of muscimol (Fig. 7h), indicating that the effect was receptor-mediated. Overall, the significant change in PPR induced by muscimol showed the presence of GABA_A_ receptors on low threshold primary afferent nerve terminals to all of the neurons receiving monosynaptic A fiber input in lamina III/IV.

### Muscimol modulation of the second peak is correlated with the control paired pulse ratio

**Fig. 8:**
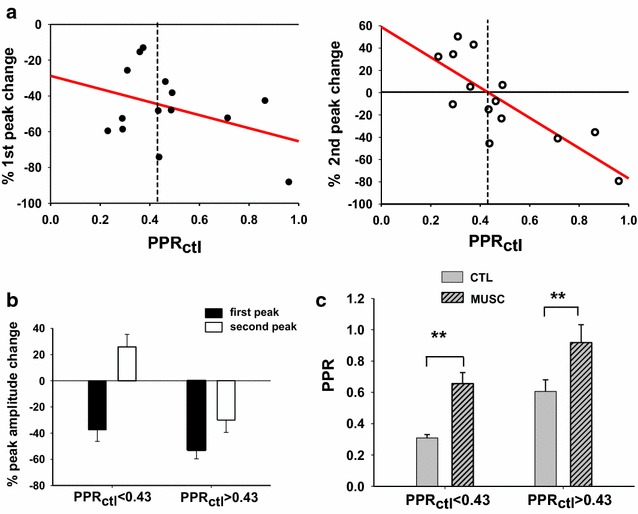
Different effects of muscimol on synapses exhibiting control PPRs that are less than or greater than 0.43. **a** Percentage changes of the first and second eEPSC peak, represented, for each individual neuron, as a function of PPR_ctl_. The first peak is always depressed (*left*), while the second peak is mainly increased in synapses with PPR_ctl_ <0.43 (n = 6) and mainly depressed in synapses having PPR_ctl_ >0.43 (n = 8) (*right*). The regression line is represented for both samples of data. **b** Average values of percentage peak changes obtained from the data shown in **a**. **c** Muscimol causes a significant increase of PPR in both synapse populations (PPR_ctl_ <0.43: paired t test, p = 0.009, n = 6; PPR_ctl_ >0.43: paired t test, p = 0.003, n = 8)

While using GABA_A_ receptor antagonist, we detected a subset of A fiber synapses in lamina III/IV with presynaptic inhibition of the second eEPSC and while using GABA_A_ receptor agonist, all A fiber synapses showed presynaptic inhibition of the first eEPSC. This raised the question of whether, in the presence of muscimol, there was evidence for a change in the second eEPSC peak in the subset of synapses likely to have functioning GABAergic synaptic input. Because these synapses had PPR < 0.43, we tested whether a muscimol-induced change in the second peak was correlated with PPR. We found that while the change in amplitude of the first peak was not correlated with PPR (Pearson correlation test, r squared = 0.15, P = 0.17, n = 14), a change in the second peak was correlated (Pearson correlation test, r squared = 0.63, P = 0.007, n = 14) (Fig. 8a). Specifically, the majority of synapses with PPR <0.43 showed an actual increase in amplitude of the second peak in response to muscimol, while those with more modest depression (PPR > 0.43) showed a decrease of the second peak (Fig. 8a, b). At synapses with PPR <0.43, the depression of the first response and the enhancement of the second drove a strong overall increase in PPR in the presence of muscimol (Fig. 8c). However, even the synapses with a depressed second response and PPR >0.43 had a significant increase in PPR (PPR_ctl_ < 0.43: paired t test, p = 0.009, n = 6; PPR_ctl_ > 0.43: paired t test, p = 0.003, n = 8; Fig. 8c). The synapses with low PPR and muscimol elevation of the second response amplitude are expected to strongly overlap with the gabazine sensitive synapses.

## Discussion

We investigated GABA-mediated negative feedback onto the central terminals of low threshold afferent fibers synapsing with lamina III/IV neurons in the rat spinal cord. We observed transient and precise timing of this activity dependent control of transmitter release that is superimposed upon a gabazine insensitive, and thus GABA_A_ receptor independent, synaptic depression with more sustained temporal characteristics. Because many of these low threshold fibers are likely to be activated by light touch [24], this feedback is predicted to mold touch sensation and even, as discussed below, contribute to mechanical allodynia.

### Evidence that gabazine relief of synaptic depression represents a presynaptic action of GABA

**Fig. 9:**
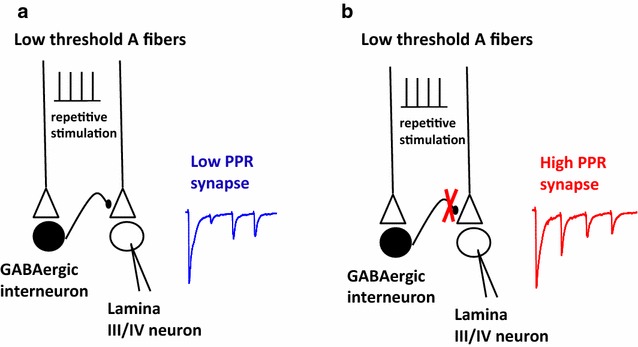
Afferent driven release of GABA onto low threshold afferent terminals can suppress strong excitatory drive in dorsal horn circuitry. **a** EPSCs evoked by strong excitatory drive (i.e., a four stimuli train in our experiments) causes strong depression of the second EPSC. **b** When GABA_A_-mediated presynaptic inhibition is absent, as is thought to occur in some chronic pain states, the strong depression on the second EPSC is relieved and the overall level of excitability in dorsal horn circuits is increased

We took several approaches to establishing that the enhanced, gabazine-sensitive depression of the second eEPSC amplitude in the PPR <0.43 synapses was due to a presynaptic effect. First, we analyzed the effects of the agonist muscimol and the antagonist gabazine on PPR. In the presence of GABA_A_ agonist and antagonist, PPR was enhanced. While not definitive, this suggests a functional presynaptic presence of GABA_A_ receptors [25]. Secondly, gabazine was able to relieve depression of the second response in a train when recordings were made with intracellular CsF, which inhibits ion flow through postsynaptic GABA_A_ receptors, indicating that postsynaptic GABA_A_ receptors were not shunting the second eEPSC. Thirdly, when glycine receptors expressed on lamina III/IV neurons were blocked by strychnine, the second eEPSC was unaffected, confirming that postsynaptic shunting was not involved in the gabazine sensitive depression of the second response. Taken together, our data constitute strong support that the gabazine sensitive enhancement of PPR reflects a response of the primary afferent terminals to synaptically released GABA (Fig. 9).

The effect of gabazine on paired pulse depression reported here is similar to the effect of bicuculline on paired pulse depression studied in vivo [10]. In the latter study, mechanical stimuli were applied to the receptive field or electrical stimulation was applied to the group II and III hair afferents directly, while recording from dorsal horn neurons. The strong depression of the response to the second stimulation was attenuated by bicuculline in a paired pulse protocol, yet, even in bicuculline, the second response remained partially depressed [10], similar to our gabazine sensitive synapses. We have extended the study of GABAergic feedback inhibition onto peripheral A fibers by stimulating with a four pulse train. Interestingly, we did not observe gabazine sensitive feedback inhibition on the third and fourth eEPSCs. This indicates that GABAergic presynaptic inhibition is precisely timed to suppress transmitter release only early in a burst of A fiber action potentials stimulated by peripheral sensory input.

### Synaptic depression includes at least two components at a subset of synapses

At all primary afferent synapses tested, there was a strong paired pulse depression in the absence of any drugs. A comparable level of depression affected the amplitudes of all three eEPSC following the first eEPSC when recorded in the presence of gabazine. In addition, at a large subset of synapses (with a PPR <0.43), there was a further significant depression of only the second response and this latter was relieved by gabazine. We did not investigate the mechanism underlying the gabazine insensitive synaptic depression. It is most likely to be activity-dependent synaptic depression [26]. However it could have been driven by other transmitters or neuromodulators accumulating near the terminals.

The GABA responsible for the gabazine-sensitive component of synaptic depression is likely to be released from synaptically driven GABAergic interneurons. We believe that the first stimulus of the stimulation train activates GABAergic neurons synapsing onto the A fiber terminal at the synapse under study (Fig. 9a). The GABA inhibits glutamate release by depolarization dependent inactivation of sodium and calcium voltage dependent-channels and/or by shunting the presynaptic membrane [5]. The fact that the second response is affected by gabazine while the first peak is unaltered confirms that GABA is released only following the activation of a synaptic circuit after the first stimulus. A different mechanism seems to be involved in the presynaptic inhibition of primary afferents mediated by GABA_B_ receptors. As shown by Yang and Ma [27], application of a GABA_B_ receptor antagonist causes the increase of the first peak and the decrease of PPR in evoked EPSCs recorded from lamina II neurons, suggesting, in this case, the presence of a tonic effect of GABA.

Even though only a subset of synapses showed enhanced depression of the second response due to synaptically released GABA, all primary afferent-mediated synapses tested were muscimol sensitive, indicating the presence of GABA_A_ receptors on or near all afferent central terminals tested. This may suggest that all of the sensory central synapses in our experiments normally have axo-axonic GABAergic terminals but that the circuitry required to drive GABA release has been cut away in some of the spinal cord slice preparations. Consistent with our physiological findings about muscimol sensitivity, most of the Aβ and Aδ hair follicle terminals receive GABAergic axo-axonic inputs [28]. Furthermore, GABA_A_ receptor subunits are expressed on the Aβ and Aδ fiber terminals in this region [29]. In lamina III/IV, the presynaptic terminals at these axo-axonic synapses mostly co-express both GABA and glycine [30]. We tested whether synaptic depression had a component mediated by glycine by testing sensitivity of PPR to strychnine. Even at synapses with PPR <0.43, strychnine did not have an impact on synaptic depression. Yet gabazine did increase PPR when tested on those same synapses. Thus glycine did not cause presynaptic inhibition even if it was co-released with GABA.

### The transient impact of GABAergic presynaptic inhibition

It is interesting to consider why synaptic depression was partially relieved by gabazine in only the second response in a train of four. Similarly, in our current clamp recordings, why was the number of action potentials in response to the second input the one most strongly modulated? The lack of gabazine effect on the third and fourth responses suggests a lack of GABA receptor activation. Thus either GABA_A_ receptors on the A fiber central terminals are desensitized, due to prior activation during the second response, or alternatively, GABA is no longer released. Block of GABA release onto afferent terminals is expected because A fiber afferent drive onto all the neurons we tested (presumably including the GABAergic neurons) is strongly depressed after the first response (Fig. 9a). Therefore the inhibitory neuron itself is less likely to be driven above action potential firing threshold in which case it will not release GABA. In either case, desensitization or blocked GABA release, GABA-mediated presynaptic inhibition is strongly diminished after the second eEPSC.

The correlation of low PPR (<0.43) under control conditions and the enhancing effect of muscimol on the peak of the second response raises the interesting question of what underlies this impact of PPR on muscimol action. After prolonged exposure to muscimol, the presynaptic receptors are expected to be partially desensitized [31]. In this population of neurons with low PPR, the second response amplitude is normally controlled by synaptically released GABA. It may be that muscimol binds to the presynaptic GABA_A_ receptors causing depression of the first peak but subsequently blocks binding of endogenous, synaptically released GABA. In this way, muscimol might effectively be acting like an antagonist to the synaptically released GABA during the second response. If so, muscimol would produce a disinhibition of the terminal to the second stimulus, leading to enhancement of the second response amplitude but only at the synapses with low PPR (<0.43).

### Implications for touch and tactile allodynia

A clear, functional role for presynaptic GABA control of transmitter release from primary afferents has recently been elegantly demonstrated in the spinal cord ventral horn [32]. When GABAergic neurons terminating on sensory afferent nerve terminals in the ventral horn were conditionally and selectively deleted, oscillations in limb trajectory during a reach movement became apparent [32]. This occurred in the continued presence of postsynaptic inhibition. In the dorsal horn, it has been proposed that presynaptic GABAergic inhibition may provide a mechanism for gating or suppressing tactile input [8]. In our experiments, the effect of GABA release onto low threshold afferents was transient and highly restricted to the beginning of a train of afferent action potentials. This pattern of presynaptic inhibition mediated by GABA_A_ receptors predicts a high temporal precision of synaptic suppression during the earliest part of an afferent response to touch. Because many touch receptors have the highest frequency of firing at the beginning of the response to a mechanical stimulus [33], this presynaptic inhibition of transmitter release is expected to distinctly impact the initial component of touch sensation. It might be expected to suppress the response of dorsal horn neurons to the early afferent drive following a mechanical stimulus, possibly decreasing touch detection threshold but also providing a mechanism to modulate that threshold.

In some studies, low threshold afferents are implicated in neuropathic pain [34]. A number of recent models of neuropathic pain suggest a critical involvement of low threshold input to deep dorsal horn and a polysynaptic pathway to lamina I or deep dorsal horn pain-associated projection neurons [12, 17, 35, 36]. Consistent with low threshold fiber involvement in mechanical allodynia is the ability of delta opioid receptor (DOR) agonists to be effective against nerve injury induced mechanical allodynia. We have previously shown that some low threshold A fibers in mice express DOR and that DOR agonist can depress release from these afferents [37]. These low threshold afferent synapses onto lamina III/IV neurons are the same population used in our study here showing transient synaptic GABAergic depression of transmitter release. This raises the possibility that GABA and endogenous activation of DOR can both act to suppress glutamate release from low threshold afferents putatively involved in both touch and mechanical allodynia.

## Conclusion

Increasingly data are showing a major role of GABA mediated presynaptic inhibition in the processing of sensory information and in the integration between sensory and motor activity in the spinal cord. Only recently, using genetic approaches, there has been improved understanding about the relative contribution of the GABAergic presynaptic versus postsynaptic inhibition in gating sensory input to the spinal cord. Our results identify a specific role of presynaptic GABA_A_ receptors in the enhancement of synaptic depression during repetitive activation of low threshold afferent fibers. Thus these receptors are able to affect neuronal excitability and sensory coding in the spinal cord dorsal horn.

## Methods

### Ethical approval

All experiments were conducted with the approval of the Columbia University Institutional Animal Care and Use Committee and of the Italian Ministry of Health, in accord with the Guide for the Care and Use of Laboratory Animals and the EU and Italian regulations on animal welfare.

### Spinal cord slice preparation

Postnatal Sprague–Dawley rats of either sex (P17–P23) were used for these experiments. At this age lamina III/IV neurons are still visible and accessible for patch-clamp recording, while GABA_A_ receptors expressed in dorsal horn exhibit properties similar to those observed in adult animals [38, 39]. Animals were anesthetized with isoflurane and decapitated, the spinal cord and vertebrae were rapidly removed and placed in ice-cold dissecting Krebs’ solution (composition in mM:125 NaCl, 2.5 KCl, 1.25 NaH_2_PO_4_, 26 NaHCO_3_, 25 glucose, 6 MgCl_2_, 1.5 CaCl_2_, and 1 kynurenic acid, pH 7.4, 320 mOsm), bubbled with 95 % O_2_, 5 % CO_2_. The lumbar spinal cord was isolated, embedded in an agarose block (low melting point agarose 3 %, Invitrogen, USA), and transverse slices (400–500 µm thick) were obtained using a vibrating microtome (WPI, USA). Slices were incubated in oxygenated incubation Krebs’ solution (same as dissecting but without kynurenic acid) at 35 °C for 1 h and used for recording.

### Patch-clamp recording and dorsal root stimulation

Patch-clamp recording in whole-cell configuration was performed on visually identified lamina III/IV neurons at room temperature. Neurons were visualized using a Zeiss Axioskop microscope, fitted with Nomarski optics and connected to a camera (Dage-MTI, USA). Slices were perfused at 2 ml/min with recording Krebs’solution (composition in mM:125 NaCl, 2.5 KCl, 1.25 NaH_2_PO_4_, 26 NaHCO_3_, 25 glucose, 1 MgCl_2_, and 2 CaCl_2_, pH 7.4, 320 mOsm). For recordings in voltage-clamp, thick-walled borosilicate pipettes, having a resistance of 3–5 MOhm, were filled with a solution having the following composition (in mM): 130 cesium methanesulfonate, 10 sodium methanesulfonate, 10 EGTA, 1 CaCl_2_, 10 HEPES, 5 lidocaine *N*-ethyl bromide quaternary salt-Cl, 2 MgATP, pH adjusted to 7.2 with CsOH, osmolarity 300 mOsm. CsF intracellular solution had the following composition (in mM): 135 cesium fluoride, 5 cesium chloride, 5 EGTA, 10 Hepes, 2 lidocaine *N*-ethyl bromide quaternary salt-Cl, pH adjusted to 7.2 with CsOH, osmolarity 290 mOsm. Recordings in current-clamp were obtained using a potassium-based intracellular solution with the following composition (in mM): 120 K-methane-sulfonate, 10 NaCl, 10 EGTA, 1 CaCl_2_, 10 HEPES, 5 ATP-Mg, pH adjusted to 7.2 with KOH, osmolarity 300 mOsm. Voltage-clamp recordings were obtained at −70 mV, while in current-clamp the membrane potential was maintained between −60 and −65 mV. Junction potential was corrected after recording. Data were recorded and acquired using a Multiclamp 700A amplifier and pClamp 9 software (Molecular Devices, USA). Sampling rate was 10 kHz, and data were filtered at 2 kHz. Lamina III/IV neurons expressing NK1 receptors (NK1R+) were identified by incubating slices with 50 nM tetramethylrhodamine conjugated substance P (TMR-SP, Anaspec, USA) for 20 min at room temperature, and washed for 1 h before recording [40].

The dorsal root attached to each slice was stimulated using a suction electrode (stimulus intensities: 25–50 μA, duration: 0.1 ms). Monosynaptic EPSCs mediated by low threshold primary afferent fibers were identified by recording 20 consecutive traces at 20 Hz [12]. Experiments on evoked synaptic responses were performed only on neurons where the EPSC was clearly visible at each trace and temporally linked to the stimulus artefact. Neurons exhibiting a large polysynaptic component shunting the subsequent EPSC were not included in this study.

### Data analysis

Data were analyzed off-line using pClamp9 software. EPSC peak amplitudes were determined in a constant 2 ms window. Mean peak amplitudes and paired pulse ratios (PPRs) were calculated from 5–6 averaged traces in the different experimental conditions. Graphs were obtained using Sigmaplot 11 (SPSS, USA) and statistical analysis was performed using GraphPad (GraphPad software, USA). Data are expressed as mean ± SE.
